# Armchair Janus MoSSe Nanoribbon with Spontaneous Curling: A First-Principles Study

**DOI:** 10.3390/nano11123442

**Published:** 2021-12-19

**Authors:** Naizhang Sun, Mingchao Wang, Ruge Quhe, Yumin Liu, Wenjun Liu, Zhenlin Guo, Han Ye

**Affiliations:** 1 State Key Laboratory of Information Photonics and Optical Communications, Beijing University of Posts and Telecommunications, Beijing 100876, China; snz@bupt.edu.cn (N.S.); quheruge@bupt.edu.cn (R.Q.); microliuyumin@hotmail.com (Y.L.); jungliu@bupt.edu.cn (W.L.); 2 Centre for Theoretical and Computational Molecular Science, Australian Institute for Bioengineering and Nanotechnology, The University of Queensland, St Lucia, QLD 4072, Australia; mingchao.wang@uq.edu.au; 3 Mechanics Division, Beijing Computational Science Research Center, Beijing 100193, China; zguo@csrc.ac.cn

**Keywords:** Janus TMD nanoribbon, nanotube, spontaneous curling, density functional theory, molecular dynamics

## Abstract

Based on density functional theory, we theoretically investigate the electronic structures of free-standing armchair Janus MoSSe nanoribbons (A-MoSSeNR) with width up to 25.5 nm. The equilibrium structures of nanoribbons with spontaneous curling are obtained by energy minimization in molecular dynamics (MD). The curvature is 0.178 nm^−1^ regardless of nanoribbon width. Both finite element method and analytical solution based on continuum theory provide qualitatively consistent results for the curling behavior, reflecting that relaxation of intrinsic strain induced by the atomic asymmetry acts as the driving force. The non-edge bandgap of curled A-MoSSeNR reduces faster with the increase of width compared with planar nanoribbons. It can be observed that the real-space wave function at the non-edge VBM is localized in the central region of the curled nanoribbon. When the curvature is larger than 1.0 nm^−1^, both edge bandgap and non-edge bandgap shrink with the further increase of curvature. Moreover, we explore the spontaneous curling and consequent sewing process of nanoribbon to form nanotube (Z-MoSSeNT) by MD simulations. The spontaneously formed Z-MoSSeNT with 5.6 nm radius possesses the lowest energy. When radius is smaller than 0.9 nm, the bandgap of Z-MoSSeNT drops rapidly as the radius decreases. We expect the theoretical results can help build the foundation for novel nanoscale devices based on Janus TMD nanoribbons.

## 1. Introduction

Janus MoSSe, an emerging member of transition metal dichalcogenide (TMD), has aroused tremendous scientific and technological attention because of its out-of-plane mirror asymmetry. Lu et al. stripped the top layer of S atoms with hydrogen plasma and substituted them with hydrogen atoms, and the subsequent thermal selenization led to the replacement of H atoms by Se to form stable monolayer of MoSSe [1]. In addition, Zhang et al. successfully synthesized single layer MoSSe by controlled sulfurization of the top atomic layer of MoSe_2_ while retaining the entire bottom Se atomic layer [2]. Since then, interest in exploiting the physical properties as well as applications of various Janus TMD-based nanostructures has intensified. Its two-dimensional (2D) structures, such as the monolayer, bilayer, and lateral heterostructure have been widely studied in growth mechanism [3], dipole moments [4], electronic structures [5,6,7,8,9,10,11,12,13], magnetic properties [14,15], phonon transport [16], piezoelectricity [17] and valleytronics [18,19]. Owing to the unique physical and chemical properties, Janus MoSSe structures has many potential applications in field-effect transistors [20], photocatalysts [21,22,23,24,25], optoelectronic devices [25,26,27,28,29,30,31,32], lithium-ion batteries [33], molecular sensors [34] and so on.

In addition to the 2D Janus TMDs, its one-dimensional (1D) derivatives, such as nanoribbons [35], are regarded as promising candidates for next-generation nanoelectronics. Wang et al. have investigated the magnetic properties of zigzag Janus MoSSe nanoribbons that could be modulated by the strain and external electric fields [36]. Due to the out-of-plane mirror symmetry breaking, a significant increase of magnetic moment in zigzag Janus MoSSe nanoribbons was demonstrated [37]. Zheng et al. found non-metal edge modification can affect the magnetic moment of zigzag Janus MoSSe nanoribbons and also can induce charge redistribution and tune the electrical characteristics of armchair Janus MoSSe nanoribbons [38]. Very recently, Hao et al. have explored the edge elasticity of Janus MoSSe nanoribbons and its effect on flexoelectric response. They further found that their width has a decisive influence on the out-of-plane flexoelectronic coefficient [39].

Experimentally, tightly scrolled TMD nanotube can be produced from monolayer TMD flakes with assistance of ethanol solution [40]. In contrast to traditional TMD nanotube, Janus TMD nanotubes are more stable owing to their small curvature energy penalty [41]. The mechanical bending, as a unique attribute of thin 2D materials, can be used to control the charge localization of top valence bands, as well as electronic conductivity and Fermi-level shift in MoS_2_ nanoribbons [42]. Meanwhile, the distinctive feature of planar Janus TMD monolayer is that the atomic asymmetry generates intrinsic strain. Demonstrated by molecular dynamics (MD) simulations, the strain relaxation allows energetically favorable spontaneous curling and even enables the formation of tube-like nanostructures [43,44]. Theoretical studies reported that the electronic properties of Janus MoSSe nanotubes, such as their bandgaps, are dependent on their diameter and chirality which can be tuned by strains [45,46,47,48]. Moreover, the reduction of semiconducting material symmetry (e.g., bending) also provides an alternative path to the investigation of the photogalvanic effect (PGE). It has been demonstrated that the PGE photocurrent obtained in 1D WS_2_ nanotubes is several orders of magnitude larger than that obtained in planar 2D WS_2_ monolayers [49].

Although efforts have been made for theoretical investigations on properties of Janus TMD nanoribbons and nanotubes, the structures considered in literature (below 120 atoms) were not large enough to demonstrate a full picture of curling behavior and spatial localization of carriers [36,37,38,39,41,45,47,48]. For example, considering the curvature, the nanotubes spontaneously formed by curling of Janus armchair MoSSe nanoribbon comprises more than 600 atoms. The gap of size is obvious. In this paper, we adopt MD, finite element method (FEM) and analytical solution to explore the spontaneous curling behaviors of armchair Janus MoSSe nanoribbons (denoted as A-MoSSeNRs). The Kohn-Sham density functional theory (KS-DFT) based on Chebyshev filtering is used to study the electronic band structure and the real-space wave function of electrons in large-scale A-MoSSeNRs and zigzag MoSSe nanotubes (denoted as Z-MoSSeNTs). The largest models of nanoribbon and nanotube consist of 477 and 654 atoms, respectively.

## 2. Materials and Methods

Firstly, we perform geometry optimization of Janus MoSSe primitive cell using Vienna ab initio simulation package (VASP, version5.4, VASP Software GmbH, Vienna, Austria) with the projector augmented wave (PAW) potentials [50,51]. Perdew-Burke-Ernzerhof (PBE) form of the generalized gradient approximation (GGA) [52] is adopted as exchange-correlation functional. The van der Waals (vdW) correction to the GGA functional is included by adopting the DFT-D3 method of Grimme [53,54]. An energy cutoff is set as 520 eV. Vacuum layer with 20 Å thickness is introduced to avoid the out-of-plane periodic interaction. The Brillouin zone of the primitive cell is sampled with a 17 × 17 × 1 k-points mesh by Monkhorst-Pack scheme [55]. The structures are fully relaxed until convergence criteria are reached, i.e., 10^−6^ eV for energy and 0.01 eV/Å for forces on each atom. The optimized lattice constants, heights of Mo-S layer (*h_Mo-S_*) and Mo-Se layer (*h_Mo-Se_*) in Janus MoSSe monolayer are 3.228 Å, 1.533 Å and 1.708 Å respectively, which are in agreement with MD simulation results [44].

**Figure 1:**
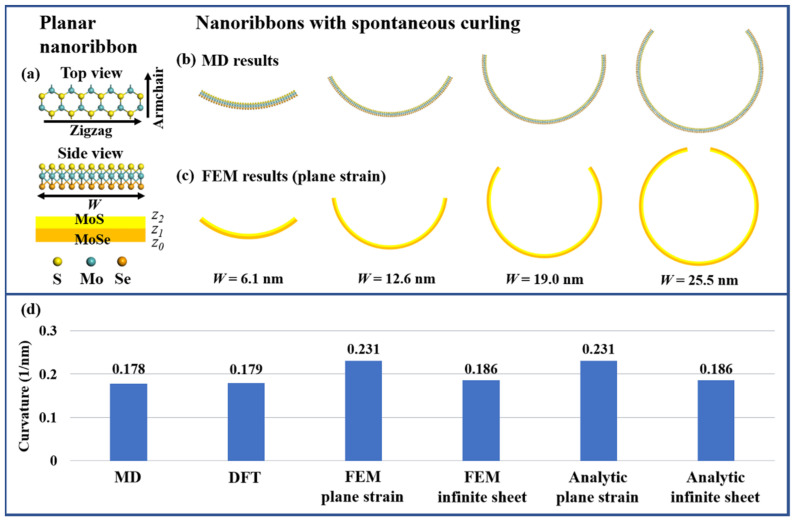
(**a**) Top and side views of atomic A-MoSSeNR structure and corresponding continuum model. (**b**) Equilibrium structures of A-MoSSeNRs obtained by MD simulations. (**c**) Equilibrium structures of A-MoSSeNRs obtained by FEM. (**d**) Curvatures obtained by MD, DFT, FEM and analytical solutions.

The primitive cell of Janus MoSSe monolayer is expanded along zigzag direction, and periodic condition is applied in the armchair direction to construct Janus armchair MoSSe nanoribbons (A-MoSSeNRs). The two edges of the nanoribbons are ensured symmetrical by deleting redundant atoms. The top and side view of A-MoSSeNR geometries are shown in Figure 1a. To explore the equilibrium structures of large-scale A-MoSSeNRs, MD simulation is performed using LAMMPS [56]. For complicated TMD alloy and heterostructures, Stillinger-Weber (SW) potential [57] has been recently developed and is adopted to describe the atomistic interactions in Janus TMDs. Starting from the planar configuration, the atoms in A-MoSSeNRs are allowed to relax by using the conjugate gradient algorithm. Force tolerance is set as 10^−10^ eV/Å. The curled nanoribbons obtained from structural relaxation are taken as equilibrium structures in the following DFT calculations. Moreover, to clearly capture the spontaneous curling and consequent sewing process of large nanoribbons to form nanotubes, a 35.19 nm wide planar nanoribbon is relaxed under NVT ensemble (i.e., constant atom, volume and temperature) for 5 ns (with the time step of 1 fs) at 0.1 K [58]. At higher temperatures, additional vibration and distortion would be introduced and the whole process would happen within a shorter time.

The first-principles calculations of armchair-oriented nanoribbons and nanotubes are carried out by Real space Electronic Structure Calculator (RESCU, version2.2, HZWTech, Shanghai, China), which is a powerful computational solver based on Kohn-Sham density functional theory with Chebyshev filtering [59]. The generalized gradient approximation (GGA) is used for the exchange-correlation potential by Perdew, Burke and Ernzerhof (PBE). Due to the presence of the heavy element Mo, spin-orbit coupling (SOC) effects are included in all calculations. A linear combination of atomic orbital (LCAO) method is used to expand physical quantities, and the standard norm-conserving pseudopotentials are used to define the atomic core states. The double zeta polarization functions (DZP) basis sets are adopted. A vacuum spacing set as 20 Å along the y-direction is used to keep the interlayer coupling negligible, and k-point mesh of 1 × 9 × 1 in the Brillouin zone is used to ensure convergence. The criteria for total energy and charge density are 10^−5^ Hartree and 10^−5^ e, respectively.

## 3. Results and Discussion

Initial free-standing planar A-MoSSeNRs are relaxed in MD simulations to obtain equilibrium structures based on energy minimization, as plotted in Figure 1b. In this relaxation, spontaneous curling can be observed along zigzag direction and with S (Se) atoms appearing in the inner (outer) circle. The curvature is 0.178 nm^−1^, as measured in the transition metal atomic layer. Interestingly, this value is almost constant at different places in one nanoribbon and regardless of nanoribbon width (marked as *W*). In this sense, the curling property of A-MoSSeNR, such as curvature, is mainly determined by the material properties. As a validation, we perform a geometry optimization of a relatively narrow (20 periods) A-MoSSeNR in VASP. The curvature determined from DFT is 0.179 nm^−1^, showing good agreement with our MD result. To further explore the origin of spontaneous curling, we resort to FEM and analytical solution based on continuum elastic theory. The geometry of A-MoSSeNR is modeled by putting a MoS sublayer on a MoSe sublayer with simple 2D geometry while the atomic asymmetry in Janus structure is modeled by two equivalent misfit strains [43]. It should be noted this is an analogy to construct Janus MoSSe with a layered model which is pervasively adopted for mismatched epitaxial systems (InAs/GaAs and Ge/Si). In FEM, two misfit strains, $\varepsilon_{MoS}=(a_{MoS_{2}}-a_{MoSSe})/a_{MoS_{2}}$ and $\varepsilon_{MoSe}=(a_{MoSe_{2}}-a_{MoSSe})/a_{MoSe_{2}}$, are imposed into the corresponding layer as initial strain and then relaxed by FE solver to determine the equilibrium structure with plane strain assumption. The curled FEM models are plotted in Figure 1c. Moreover, this simplified 2D two-layered bending model can be described by an analytical treatment [60,61]. The curvature is expressed as [60]

(1) $$\kappa =\frac{3\sum_{i=1}^{2}E_{i}^{\prime }h_{i}(z_{i}+z_{i-1}-2z_{b})(c-\eta_{i}\varepsilon_{i}^{0})}{2\sum_{i=1}^{2}E_{i}^{\prime }h_{i}[z_{i}^{2}+z_{i}z_{i-1}+z_{i-1}^{2}-3z_{b}(z_{i}+z_{i-1}-z_{b})]}\;\begin{array}{l} c=\frac{\sum_{i=1}^{2}E_{i}^{\prime }h_{i}\eta_{i}\varepsilon_{i}^{0}}{\sum_{i=1}^{2}E_{i}^{\prime }h_{i}} \end{array}\;z_{b}=\frac{\sum_{i=1}^{2}E_{i}^{\prime }h_{i}(z_{i}+z_{i-1})}{2\sum_{i=1}^{2}E_{i}^{\prime }h_{i}}$$

where subscript *i* denotes the layer number from bottom to top, *h_i_* is the height of *i*th layer, *z_i_* is as marked in Figure 1a and $\varepsilon_{i}^{0}$ is the misfit strain in *i*th layer. For plain strain assumption, $E_{i}^{\prime }=E_{i}/(1-\nu_{i}^{2})$ and $\eta_{i}^{}=1+\nu_{i}^{}$, while for infinite sheet assumption, $E_{i}^{\prime }=E_{i}/(1-\nu_{i}^{})$ and $\eta_{i}^{}=1$. *E =* 299 GPa and ν=0.245 [44] are Young’s modulus and Poisson ratio, respectively. Detailed derivation process can be found in Reference [60]. The curvatures evaluated by MD, DFT, FEM and analytic solution are summarized in Figure 1d. The qualitatively consistent results confirm that the relaxation of intrinsic strain in Janus structure acts as the driving force of spontaneous curling. In our previous work, the curvature of hexagonal Janus MoSSe quantum dots was 0.149 nm^−1^ and this lower value comes from more flexible free edges in quantum dots for strain relaxation, compared with A-MoSSeNR.

**Figure 2:**
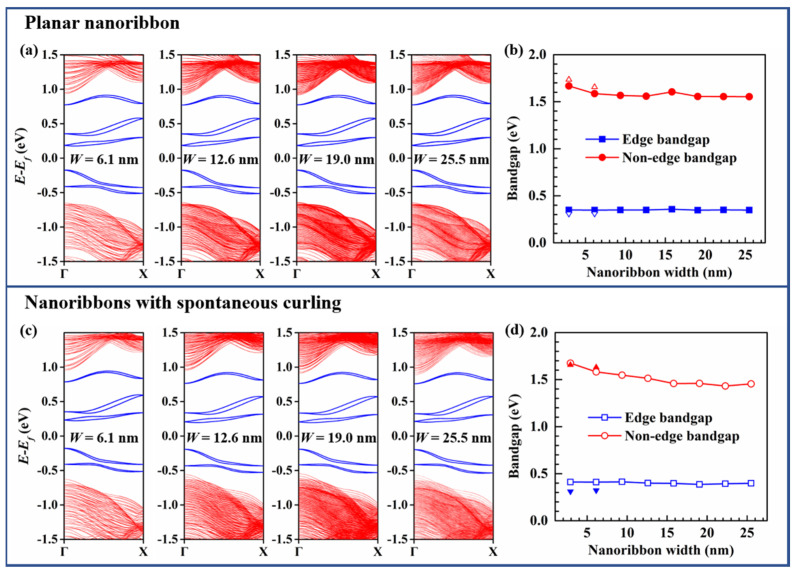
(**a**) Band structures of planar A-MoSSeNRs. The bands highlighted in blue are 2-fold degenerate edge-state bands. (**b**) Relation between bandgap of planar A-MoSSeNR and nanoribbon width. (**c**) Band structures of spontaneously curled A-MoSSeNRs. (**d**) Relation between bandgap of curled A-MoSSeNR and nanoribbon width. The triangle symbols are calculated by VASP.

Here we investigate the electronic structure of sixteen A-MoSSeNRs, including eight planar and eight curled nanoribbons. The width ranges from 6.1 nm to 25.5 nm. Since our nanoribbon models are relatively large and we intentionally focus on the curling effect, the curled nanoribbons are directly exported from LAMMPS without further optimization on the edges. Consistent with previous studies [38], there are twenty edge states in the band structures of A-MoSSeNR, including the 2-fold-degenerate bands and the bands split by the SOC effect. Both planar and curled A-MoSSeNRs without passivation are indirect-gap semiconductors (Figure 2a,c). If we focus on the non-edge states, A-MoSSeNR is a direct-gap semiconductor. Due to the axial symmetry of nanoribbons, the electronic bands of planar and curled A-MoSSeNRs are all 2-fold-degenerate. The bandgap is formed between two 2-fold-degenerate edge-state bands which is closest to Fermi level. To explore the effect of spontaneous curling, we studied the evolution of bandgaps of planar and spontaneously curled A-MoSSeNR with different widths respectively, as shown in Figure 2b,d. As the width increases, the edge bandgaps of planar and curled A-MoSSeNRs keep at the constant values of 0.34 eV and 0.41 eV. Meanwhile, the non-edge bandgap of curled A-MoSSeNR decreases from 1.67 eV with the width of 6.1 nm to 1.45 eV with the width of 25.5 nm. This indicates that the spontaneous curling effect would be beneficial to the energy shift in the non-edge electronic bandgap.

**Figure 3:**
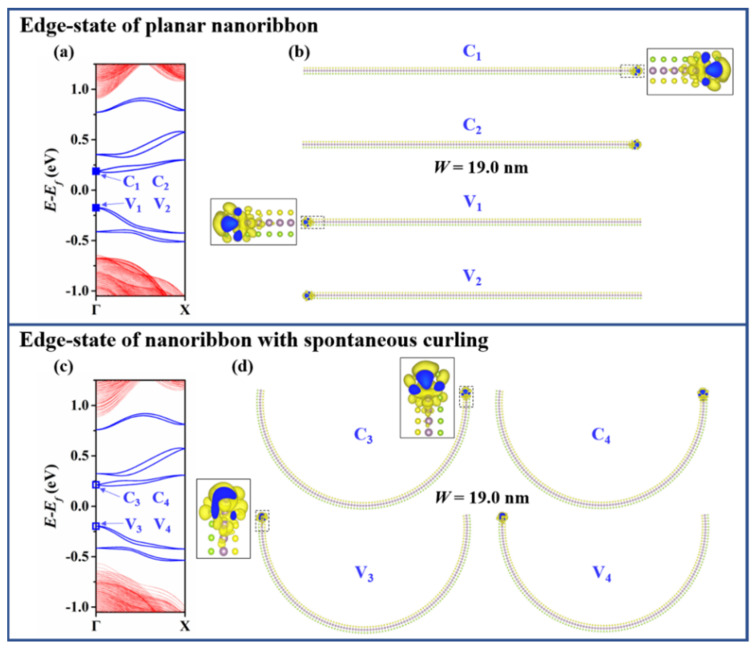
Spontaneous curling effect on edge-state of A-MoSSeNR (*W* = 19.0 nm) (**a**) Band structure of planar A-MoSSeNR. (**b**) Isosurfaces of real-space wave function of state C_1_, C_2_, V_1_ and V_2_. (**c**) Band structure of curled A-MoSSeNR. (**d**) Isosurfaces of real-space wave function of state C_3_, C_4_, V_3_ and V_4_.

**Figure 4:**
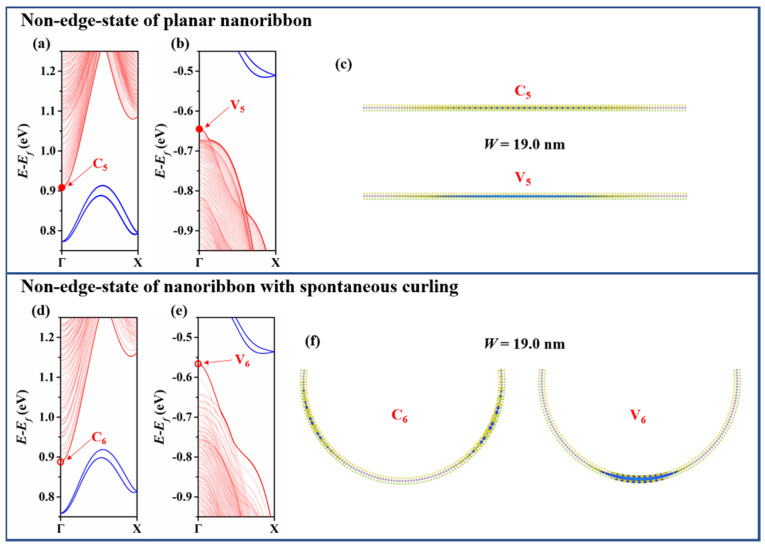
Spontaneous curling effect on non-edge-state of A-MoSSeNR (*W* = 19.0 nm) (**a**) Conduction and (**b**) valence band structure of planar A-MoSSeNR. (**c**) Isosurfaces of real-space wave function of state C_5_ and V_5_. (**d**) Conduction and (**e**) valence band structure of curled A-MoSSeNR. (**f**) Isosurfaces of real-space wave function of state C_6_ and V_6_.

To further explore the spontaneous curling effect on the charge carriers of A-MoSSeNR, we resort to the real-space wave function. Figure 3 demonstrates the real-space wave functions of edge states at Γ point. The carriers are localized at the unpassivated edges whether the nanoribbons spontaneously curl or not. The state in valence band is denoted as V, while the state in conduction band is denoted as C. It is worth mentioning that the real-space wave functions at Γ point of the two valence bands closest to Fermi level (V_1_ to V_4_) are localized on the left edge of nanoribbon, while the corresponding conduction band ones (C_1_ to C_4_) are localized on the right. If edges are passivated, the conductivity behavior would be dominated by the localization of wave functions at valence band maximum (VBM) and conduction band minimum (CBM) of non-edge-states. The partial charge densities at the top of the non-edge valence bands of A-MoS_2_NR with width less than 4 nm have been investigated [42]. Consistent with the previous study, the wave function at the top of non-edge valence bands (V_5_) is delocalized over the whole planar A-MoSSeNR. As illustrated in Figure 4, when curling occurs, the wave function at top valence band (V_6_) clearly shrinks to the central region. On the other hand, the profile of wave function at the bottom of non-edge conduction bands (C_5_) is basically identical to V_5_ in planar nanoribbon. However, the bottom conduction band (C_6_) wave function dissipates from the central region and is separated to lateral regions in nanoribbon after spontaneous curling.

**Figure 5:**
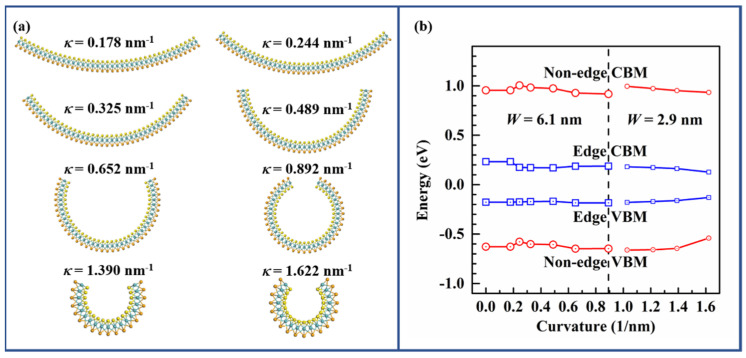
(**a**) Atomic structures of A-MoSSeNRs with curvature κ ranging from 0.178 nm^−1^ to 1.622 nm^−1^. The width of the upper six nanoribbons is 6.1 nm, and the width of lower two nanoribbons is 2.9 nm. (**b**) Evolution of CBM and VBM of A-MoSSeNR with respect to curvature.

As discussed above, spontaneous curling slightly reduces the non-edge bandgap of A-MoSSeNR and affects its real-space wave function. The curvature role in its electronic properties needs to be further discussed. As shown in Figure 5a, the A-MoSSeNR with width of 6.1 nm is adopted. One can imagine that the curvature of the nanotube with circumference of 6.1 nm is 1.03 nm^−1^, which represents the maximum curvature of such nanoribbon. The maximum curvature of 6.1 nm wide nanoribbon is thus set 0.892 nm^−1^. To demonstrate the curling effects under larger curvature, we chose nanoribbon with a smaller width of 2.9 nm, and the limit value of curvature is set 1.622 nm^−1^. Figure 5b demonstrates that the bands-edge of A-MoSSeNR has no obvious energy shift as the curvature increases when κ < 0.892 nm^−1^. Both the energy of edge-states and non-edge-states shift to the Fermi level as the curvature further increases, reflecting that larger curvature reduces the bandgap. For non-edge-states, the shift of CBM under curling is smaller than that of VBM. As reported, the edge states shift down to the non-edge valence bands at a critical curvature [42,62]. Hence the Fermi-level pinning is removed when curvature is larger than a critical value. Since we intend to probe the spontaneous curling effect in large-scale A-MoSSeNR, we do not intentionally optimize the edge atoms for reconstruction and the way to find equilibrium structures is different from the literature. As a result, similar effects are not observed.

**Figure 6:**
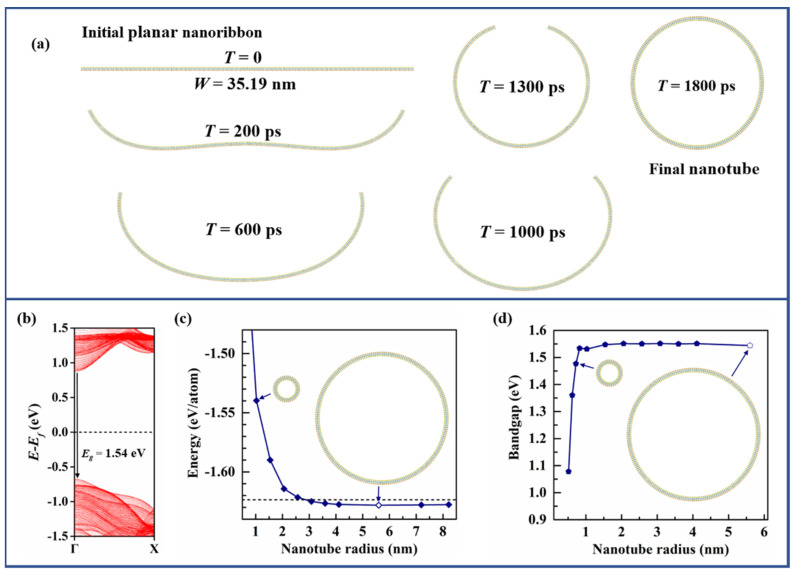
(**a**) Snapshots of spontaneous formation process of Z-MoSSeNT. (**b**) Band structure of spontaneously formed Z-MoSSeNT. (**c**) Per-atom energy of Z-MoSSeNT as a function of radius. (**d**) Bandgap of Z-MoSSeNT as a function of radius.

Finally, considering that the curvature of MoSSe nanoribbon is around 0.178 nm^−1^, we demonstrate that a 35.19 nm wide (109 unit cells in zigzag direction) A-MoSSeNR can spontaneously form a MoSSe nanotube (Z-MoSSeNT). In MD simulations, the initial flat nanoribbon is relaxed for 5 ns at temperature of 0.1 K. The snapshots of the spontaneous curling and consequent sewing process are illustrated in Figure 6a. The radius of this formed nanotube is 5.6 nm. Due to the absence of dangling bonds, the band structure has no edge-states and Z-MoSSeNT is a direct-gap semiconductor with a bandgap of 1.54 eV, as shown in Figure 6b. The spontaneously formed A-MoSSeNT possesses the lowest energy (−1.6281 eV/atom) among the configurations with various radii. As expected, when radius increases, the energy drops rapidly, reaches the lowest value and then gradually approaches the energy of planar monolayer (−1.6236 eV/atom), as shown in Figure 6c. The dependence of bandgap on radius is shown in Figure 6d. When radius is smaller than 0.9 nm, a rapid increase of bandgap as the increase of radius can be observed. This tendency agrees with References [46,48]. Then, bandgap continues to increase slowly and stabilizes at around 1.54 eV when radius is larger than 2 nm.

## 4. Conclusions

In summary, we investigate the impact of spontaneous curling on the electronic structures of large-scale free-standing A-MoSSeNRs. The non-edge bandgap of curled A-MoSSeNR varies from 1.67 eV to 1.45 eV with the increase of nanoribbon width up to 25.5 nm. Compared with planar nanoribbons, the real-space wave function at the non-edge VBM is localized in the central region. In addition, when the curvature is larger than 1.0 nm^−1^, the further increase of curvature will obviously shrink both edge and non-edge bandgaps of A-MoSSeNRs. Since the reconstruction of edge is not taken into consideration, the energy of edge-state is stable regardless of width and curvature. Moreover, we explore the spontaneous formation process of Z-MoSSeNT from A-MoSSeNR in MD simulations. The radius of this spontaneously formed nanotube is 5.6 nm, and it possesses the lowest energy compared with nanotubes with other curvatures. When the radius is smaller than 0.9 nm, the rapid reduction of Z-MoSSeNT bandgap can be observed with the decrease of radius.

## Data Availability

The data presented in this study are available on request from the corresponding author.
